# Comparison of the acute metabolic effect of different infant formulas and human milk in healthy adults: a randomized trial

**DOI:** 10.1038/s41387-021-00154-3

**Published:** 2021-04-15

**Authors:** Yasaman Shahkhalili, Cathriona Monnard, Dominik Grathwohl, Julien Sauser, Maurice Beaumont, Corinne Ammon Zufferey, Katherine Macé

**Affiliations:** 1 Société des Produits Nestlé S.A, Lausanne, Switzerland; 2 Université de Genève, Geneva, Switzerland

**Keywords:** Type 2 diabetes, Homeostasis

## Abstract

**Background/Objectives:**

Different infant formulas, varying in protein type and quantity, are available for infants who are not breastfed or are partially breastfed. Postprandial insulinemic and glycemic responses to intact vs partially hydrolyzed protein in infant formula are unclear. To compare the effect of different forms (partially hydrolyzed vs non-hydrolyzed) and levels of protein in infant formula compared with a human milk reference subgroup on insulin response in adults.

**Subjects/Methods:**

In a randomized, double-blinded, cross-over study, 35 healthy adults consumed 600 ml of three different infant formulas: Intact protein-based formula (INTACT) (1.87 g protein/100 kcal; whey/casein ratio of 70/30; 63 kcal/100 ml), partially hydrolyzed whey-based formula (PHw) (1.96 g protein/100 kcal; 100% whey; 63 kcal/100 ml), a high-protein partially hydrolyzed whey-based formula (HPPHw) (2.79 g protein/100 kcal; 100%whey; 73 kcal/100 ml) and a subgroup also consumed human milk (HM) (*n* = 11). Lipid and carbohydrate (lactose) contents were similar (5.1–5.5 and 10.5–11.6 g/100 kcal, respectively). Venous blood samples were taken after overnight fasting and at different intervals for 180 min post-drink for insulin, glucose, blood lipids, GLP-1, glucagon, and C-peptide.

**Results:**

Twenty-nine subjects (eight consuming HM) adhered to the protocol. INTACT and PHw groups had similar postprandial insulinemia and glycaemia (*C*_max_ and iAUC) that were not different from those of the HM subgroup. HPPHw resulted in higher postprandial insulin responses (iAUC) relative to all other groups (*p* < 0.001, *p* < 0.001, *p* = 0.002 for the comparison with INTACT, PHw, HM, respectively). HPPHw resulted in a higher glucose response compared to INTACT and PHw (iAUC: *p* = 0.003, *p* = 0.001, respectively), but was not different from HM (*p* = 0.41).

**Conclusion:**

This study in adults demonstrates similar postprandial insulinemia and glycaemia between INTACT and PHw, close to that of HM, but lower than HPPHw, which had a higher protein content compared to the other test milks. The findings remain to be confirmed in infants.

**Clinical trial registration:**

This study is registered at clinicaltrials.gov, identifier NCT04332510.

## Introduction

Breastfeeding is the optimal source of nutrition for infants and confers many benefits for both the infant and the mother. Despite the current recommendation of exclusive breastfeeding for the first 6 months of life^1^, surveys show that many infants receive at least some formula during the first year of life^2^. In the US, 83% of mothers initiated breastfeeding and by 6 months 57.6% continued to do so^3^, while in the UK, 81% of mothers initiated breastfeeding, which dropped to 34% by the age of 6 months^2^. Although breastfeeding is the best source of nutrition for infants, given that infants may consume some formula during their first year of life, it is important to ensure that the composition and metabolic responses to infant formula are as close as possible to breast milk.

Mature human milk (HM) contains high-quality protein in intact form (non-hydrolyzed) and the hydrolysis of HM proteins begins first in the mammary gland and continues in the term infant’s stomach^4^. The whey-to-casein ratio of HM varies depending on the stage of lactation (between 80:20 and 70:30 in early lactation and 50:50 in late lactation), with a protein concentration of ≈1.2 g/100 kcal (5% energy)^5^. The current protein concentration of formula (for infants from birth to 6 months with a minimum body weight of 2.5 kg) is 1.8–2.0 g/100 kcal (7.2–8% energy). The protein in infant formula is commonly in the form of intact protein (non-hydrolyzed) with a whey-to-casein ratio of 70:30 or partially hydrolyzed 100% whey. Partially hydrolyzed whey formula is often used in infants at risk of atopy based on a family history of atopic disease in siblings or parents^6^, although the metabolic response to such formula compared to the standard formula has not been assessed.

Diets that induce low glycaemic and insulinemic responses have been shown to exert beneficial effects on the risk factors associated with type 2 diabetes and cardiovascular disease in adults^7,8^. Unlike many carbohydrate-rich foods where a linear correlation is observed between glycaemic index (GI) and insulinemic index^9^, cow’s milk produces a considerably higher insulinemic index than is expected based on its relatively low GI^10,11^. Both infant formula and breast milk have also been shown to produce disproportionately higher insulin than glucose responses for their relatively low GI (GI = 55)^12^. The insulin response to milk is related not only to the lactose content, but also to other components such as protein and lipids^13^. Food proteins have been found to differ in their effects on glucose metabolism in humans^14,15^ and several amino acids (e.g. branch-chained amino acids) are potent stimulators of insulin secretion^16^.

In addition, the different fractions of milk protein (casein and whey) and different forms of the protein (intact vs. hydrolyzed protein) are reported to induce different insulin responses. For example, the post-prandial insulin response to whey protein is reportedly greater than that of casein^13,17^. Protein hydrolysates have also been shown to stimulate insulin secretion to a greater extent than intact protein in some^18^, but not all^19,20^ studies. It is important to bear in mind that the diets used in these studies often do not reflect the composition of milk consumed by infants during early life, which tends to be low protein, high fat, and high lactose —a low GI sugar.

Although some studies show that formula-fed infants have higher postprandial insulin secretion than breastfed infants, these studies have methodological limitations^21–23^. Furthermore, to the best of our knowledge, the effect of different forms (partially hydrolyzed vs non-hydrolyzed) of protein in infant formula compared with HM, on insulin response has not been investigated in infants or adults. Therefore, the aim of this study was to compare the effects of either intact protein (70% whey: 30% casein; INTACT), partially hydrolyzed whey protein (PHw; 100% whey) or a high-protein partially hydrolyzed whey formula (HPPHW) and HM in healthy adults. We hypothesised that insulin response was similar between INTACT and PHw formula. The recruitment of infants for this study was precluded due to the requirement for frequent blood sampling.

## Methods

### Participants

**Fig. 1:**
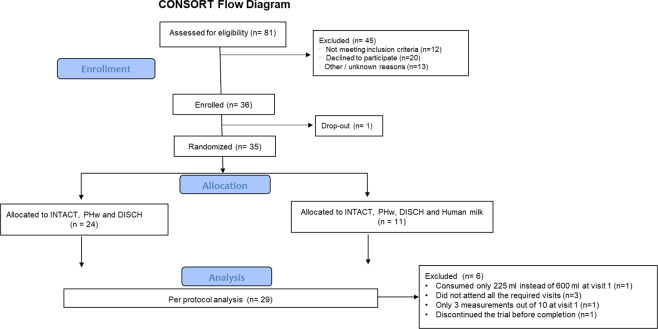
Details of participant enrolment, allocation and inclusion in the study analysis illustrated in a Consort flow diagram.

Details of the enrolment of subjects, their allocation to treatment, disposition status and how they are analysed in the trial are illustrated in Fig. 1. Participants were recruited from the Nestlé Research Center, Lausanne, Switzerland from June to October 2012. Eligibility criteria included: healthy males and females aged 20–50 years at the time of the enrolment with a BMI in the range of 19–25 kg/m^2^. Exclusion criteria were as follows: chronic or acute diseases affecting metabolism (diabetes, renal insufficiency, CVD, liver disease), dyslipidaemia, as checked using the medical questionnaire and based on a biochemical blood analysis (glucose, triglycerides, cholesterol, transaminases and Gamma-GT, CRP in fasting conditions), anaemia (erythrocytes < 4.6 T/l (male) or <4.2 T/l (women); haemoglobin Hb <13 g/dl (male) or Hb <12 g/dl (women); haematocrit Ht <40% (male) or Ht <37% (women); sera iron <0.6 mg/l or plasma ferritin <120 μg /l (male) or <60 μg/l (non-menopausal women); recent major surgery (within 3 months), history of cancer within the past year; significant weight loss during the last 3 months (>5% of body weight); regular intense (>45 min) physical activity greater than three times per week; food allergy, e.g., lactose intolerance; chronic medication use (except oral contraceptive pills); high habitual alcohol consumption (>1 drink/day or >1–2 drinks during weekends); illicit drug use, verified by urinary testing; smoking (>5 cigarettes per day); pregnant or lactating women; following a special weight reduction program /diet; have donated blood (>300 ml in the previous 3 months) or planned blood donation before the end of the study; participants who cannot be expected to comply with the study procedures, including consumption of the test products; currently participating or having participated in another clinical trial within 4 weeks prior to the beginning of this study. All participants provided written, informed consent prior to the start of the study. The study was carried out in the Nestlé Research Center/Metabolic Unit (MU) in accordance with guidelines laid down in the Declaration of Helsinki and was approved by the Commission cantonale d’éthique de la recherche sur l’être humain, Lausanne, Switzerland (protocol no. 398/11). The study is registered at clinicaltrials.gov, identifier NCT04332510.

### Study design

This study was a randomized, double blinded, mono-centre, cross-over study with four study products. The primary objective was to show that the postprandial insulin response to a partially hydrolyzed formula (PHw) is similar to that of infant formula with intact protein (INTACT). The Cmax of the insulin kinetics was the primary outcome used to demonstrate the primary hypothesis. The secondary objectives were to compare postprandial insulin and glucose responses between all formulae and HM. Based on the sample size calculation, in order to show a 20% difference with a power of 80%, *n* = 34 subjects would be required. For the sample size calculation, the within subject standard deviation was derived from another trial (internal data) with 40% difference and the alpha level of 5% was chosen. At the time the trial was conducted, we had limited access to HM, which was provided from a milk bank located in Necker Hospital, Paris, France. All HM was safe for human consumption and was provided along with a safety certificate. Thus, HM was only available for *n* = 9 subjects.

Products were coded using digit-letter-digit codes and the sequence was attributed by an internal randomization program. A biostatistician assigned the sequences and the study manager decided the final assignment to the product (e.g. code identifications “A”, “B”, “C” and “D”). The assignment to each product was shared with a dedicated person who was not involved in the study, but who prepared the products and allocated individual codes on the corresponding glass of milk in order to ensure that the subjects and study staff were blinded.

Therefore, nine participants were randomized to receive 4 test products (partially hydrolyzed whey protein formula (PHw; 1.96 g/100 kcal), intact protein formula (INTACT; 70/30 whey casein; 1.9 g/100 kcal), a formula containing partially hydrolyzed 100% whey protein at a higher concentration (HPPHW; 2.8 g/100 kcal) and HM (a sub-group as reference) and 24 participants were randomized to receive only three products: the partially hydrolyzed formula, the intact protein formula and the HPPHw formula. The average macronutrient composition of HM compared with each intervention is outlined in Table 1. The HPPHw formula contained 65% higher protein compared to the other test milk. Participants completed a maximum of 4 test days. Test days were separated by a wash-out of 2 weeks ± 2 days for men. Women were investigated during the same phase (follicular) of the menstrual cycle for all tests. Therefore, the wash-out period for women was 4 weeks ± 2 days. The total study duration for men who completed a sequence of four products was ~7 weeks, while the study duration for women who completed the sequence of four products was ~13 weeks. Prior to each study day, participants were provided instructions on how to consume a diet containing adequate carbohydrate (3 days prior). The day preceding the test, participants attended the MU and were provided with a fixed breakfast and lunch, and a fixed dinner, which they were instructed to consume at home between 20:00 and 21:00. Participants were requested to fast (no food or drink, except water) after 21:00 on the night before the test. On each study day, a volume of 600 mL of each product was ingested orally within 10 min. A test day was completed 3 h following product intake. Compliance was recorded during the study, as well as information on adverse events. Participants remained seated during the test and were permitted to read or watch TV. At the end of each experiment, participants were provided with a light lunch before leaving the MU.

**Table 1:** Energy and macronutrient composition of infant formulas and human milk per 600 ml as consumed.

	INTACT	PHw	HPPHW	Human milk^a^
Energy, kJ	1582	1582	1833	1682
Energy, kcal	378	378	438	402
Protein, g (%E)	7.08 (7.5)	7.41 (7.2)	12.24 (11)	7.2 (7.2)
Whey/casein ratio	70/30	100/0	100/0	60/40
Carbohydrate, g (%E)	43.86 (46)	43.85 (46)	45.96 (42)	42 (42)
Lipids, g (%E)	19.3 (46)	19.2 (46)	22.8 (47)	22.2 (50)

^a^Average values for the composition of human milk based on an in-house analysis of term, mature human milk.

Following the measurement of standing height and body weight, to obtain arterialized blood, a retrograde catheter (i.e. the end of the catheter placed contrary to the direction of blood flow) was inserted into one vein of the arm, then participants were asked to put their forearm in a heated box at 55 °C for 10 min before each blood sample according to the method described by Matthews et al.^24^. A saline solution was infused intravenously to keep the catheter free. Participants rested for a period of 15 min and following the rest, two baseline arterialized blood samples were taken at intervals of 30 min (time −30 and 0) in two different tubes (5 mL each) each time. Following the baseline samples, participants ingested the allocated study product (600 mL study milk) within a 10 min interval (300 mL within 5 min). The duration of intake was recorded in minutes so that participants consumed all the subsequent drinks within the same time period as the first drink during all test days. Arterialized blood samples were taken at 15 min intervals from the start of product ingestion during the first hour (time 15, 30, 45 and 60 min) and at 30 min intervals during the subsequent 2 h (time 90,120, 150 and 180 min) using two different tubes (5 mL each) each time. In total, 100 mL of blood was collected at each visit and plasma was stored at −40 °C and −80 °C prior to analysis.

### Blood measurements

Blood samples were collected into EDTA and heparinized tubes, kept on ice and centrifuged as soon as possible at 4 °C for plasma sampling. The plasma aliquots were frozen immediately and stored at –40 °C or −80 °C until further analysis. Samples were used to measure glucose, insulin, total and active GLP-1, glucagon, C-peptide, amino acids, free fatty acids and triglycerides. Upon thawing, assays were performed according to the manufacturers’ instructions: (i) insulin by using an ELISA kit (RE53171, IBL International, Germany); (ii) glucose (Siemens Dimension® clinical chemistry system); (iii) C-Peptide by ELISA kit (EZHCP-20K; Merck Millipore, Germany); (iv) Active GLP-1 by ELISA (EGLP-35K; Merck Millipore); (v) glucagon by ELISA (EZGLU-30K Merck Millipore); (vi) triglycerides (Siemens Dimension®) and (vii) free fatty acids using the ACS-ACOD method (NEFA-HR^2^ kit, Wako, Neuss, Germany).

### Statistical analysis

The predeclared primary outcome for the study was the maximal increase in the plasma concentration of insulin (Cmax). Predeclared secondary endpoints were (i) to compare postprandial insulin and glucose responses to all formulas and HM, and (ii) To compare free fatty acids, triglycerides, C-peptide, glucagon, active GLP-1 and amino acid responses. Neither the primary nor secondary endpoints changed from those that were predeclared during the course of the research or during the analyses.

Statistical analysis was performed by using a mixed model. Fixed effects were baseline, time (as a category) sex and treatment, subject was a random effect. Box-Cox transformation was applied in order to achieve approximately normally distributed residuals. Model validation was performed by visual inspection of a residuals-over-fitted-values-plot and a qq-plot. Different metabolic responses over time by product group were tested by likelihood-ratio (LR) tests. However, the asymptotic statistics of statistical tests derived from mixed models are not valid^25^. Therefore, the Null-distribution of the statistics was estimated by permutations of the product groups. Curve characteristics were estimated with the help of the predicted means after back-transformation, such as the area under the curve (AUC), the incremental area above base-line value under the curve (iAUC) and Cmax. Any reduction of AUC below baseline is represented as negative AUC (nAUC; used for free-fatty acids only). The different AUCs are divided by the time period and are therefore presented in the units of the metabolite. Statistics on the curve characteristics were also performed by permutation tests. Based on the results, AUC and iAUC were more stable and powerful than Cmax, therefore, we report results on AUC and iAUC, except for the primary comparison where Cmax was reported in line with the initial protocol. The nAUC is reported for fatty acids only.

In order to guide the interpretation of “similar” between INTACT and PHw, we made use of the European Medicines Agency (EMA) guideline on the investigation of bioequivalence. Therein, it is described that bioequivalence can be concluded when the ratio of test and reference in iAUC and Cmax are contained within the 90% confidence interval of 80 and 125%, which translates to a difference of between −20 and +25%. We consider the HM group as the reference. Therefore, we divided the differences between product groups by the mean of the HM reference group and displayed the differences as percentage.

If the LR-tests indicated different trends, we consulted the respective curve characteristics such as iAUC in order to interpret the differences.

The analyses presented are on subjects who adhered to the protocol. The mixed model was programmed with lmer from the lme4-library. The box-cox transformation was used from MASS-library. For the linear interpolation for calculating the iAUC, approxfun in the stats-library was used. For calculating tail probabilities on permutation tests, the logspline-library was used. All libraries can be found in the R statistical programming environment version 3.5.2.

## Results

### Subject characteristics

Thirty-five subjects were recruited and randomized. Six subjects had either missing test days or too many missing measurements on a test day (more than 2 measurements out of 10) or did not consume all of the allocated milk. Two adverse events defined as having a probable link to the interventions were reported: one subject randomized to receive HM, experienced nausea in response to the milk and drank only 225 ml of the assigned 600 ml; another subject experienced nausea, upper abdominal pain and headache in response to PHw formula and as a result, withdrew after visit 2. Thus, after exclusion of those who had missing data or were non-compliant, 29 subjects remained for inclusion in the analysis of the infant formulas and 8 subjects for the HM comparison. The subjects were 13 females and 16 males with a mean age of 31 years. The BMIs ranged from 19.3 to 24.9 kg/m^2^ with an average of 21.8 kg/m^2^ for females and 22.6 kg/m^2^ for males. Subject characteristics are presented in Table 2.

**Table 2:** Subject characteristics.

Variable	Total (*n* = 29)	Female (*n* = 13)	Male (*n* = 16)
Age, years	30.9 (8.7)	30.3 (9.5)	31.3 (8.3)
Height, cm	174 (8)	169 (6)	178 (7)
Weight, kg	68 (10)	63 (6)	72 (10)
BMI, kg/m^2^	22.3 (1.8)	21.8 (1.7)	22.6 (1.9)

Values are mean (±standard deviation).

### Metabolic responses

#### Insulin

Data on insulin iAUC and Cmax can be found in Table 3. Mean iAUC for HPPHW, HM, PHw, and INTACT were 18.7, 13.1, 11.9, 11.8 uIU/ml, respectively. Mean Cmax values for HPPHW, HM, PHw and INTACT were 57.2, 43.4, 40.3, 40.9 uIU/ml, respectively. The iAUC difference between PHw and INTACT was 1.5%, 90% CI −9.7 to 12.67% with respect to the mean of the HM-group (*p* = 0.95). The Cmax difference between PHw and INTACT was −2.1%, 90% CI −12.2 to 7.9% with respect of the mean of the HM-group (p = 0.81). Given that the CI are within the boundaries defined by the EMA (-20%, +25%), bioequivalence was demonstrated for iAUC and Cmax between PHw and INTACT.

**Table 3:** Metabolic responses to infant formulas, varying in type and quantity of protein, and human milk.

		INTACT *n* = 29		PHw *n* = 29		HPPHW *n* = 28		HM *n* = 8	
Insulin, μIU/ml	iAUC	11.79	(6.56)	11.92	(7.13)	18.66	(11.62)	13.10	(6.41)
	Cmax	40.88	(26.55)	40.29	(25.94)	57.24	(29.26)	43.36	(22.73)
Glucose, mmol/l	iAUC	0.24	(0.21)	0.24	(0.25)	0.45	(0.39)	0.40	(0.28)
	Cmax	5.83	(0.92)	5.86	(1.02)	6.25	(0.87)	6.14	(0.63)
Active GLP-1, pmol/l	iAUC	5.28	(3.46)	3.80	(2.28)	4.62	(2.48)	2.55	(1.24)
C-peptides, ng/ml	iAUC	0.84	(0.65)	0.83	(0.62)	1.34	(1.03)	0.90	(0.54)
Glucagon, pg/ml	iAUC	5.31	(7.16)	4.04	(5.44)	4.01	(5.78)	2.31	(3.79)
Triglycerides, mol/l	iAUC	189.8	(175.8)	183.2	(116.5)	291.6	(162.2)	181.4	(131.1)
Free fatty acids, μmol/l	nAUC	−143.4	(107.2)	−150.1	(123.0)	−165.8	(136.8)	−187.1	(102.1)
Essential AA, mol/l	iAUC	116.2	(26.4)	86.7	(28.7)	156.8	(58.0)	93.3	(37.9)

Values are mean (±standard deviation).

*INTACT* intact protein formula, *PHw* partially hydrolyzed whey protein formula, HPPHW formula; *HM* human milk, *iAUC* incremental area under the curve, *nAUC* negative area under the curve, *Essential AA* essential amino acids.

**Fig. 2:**
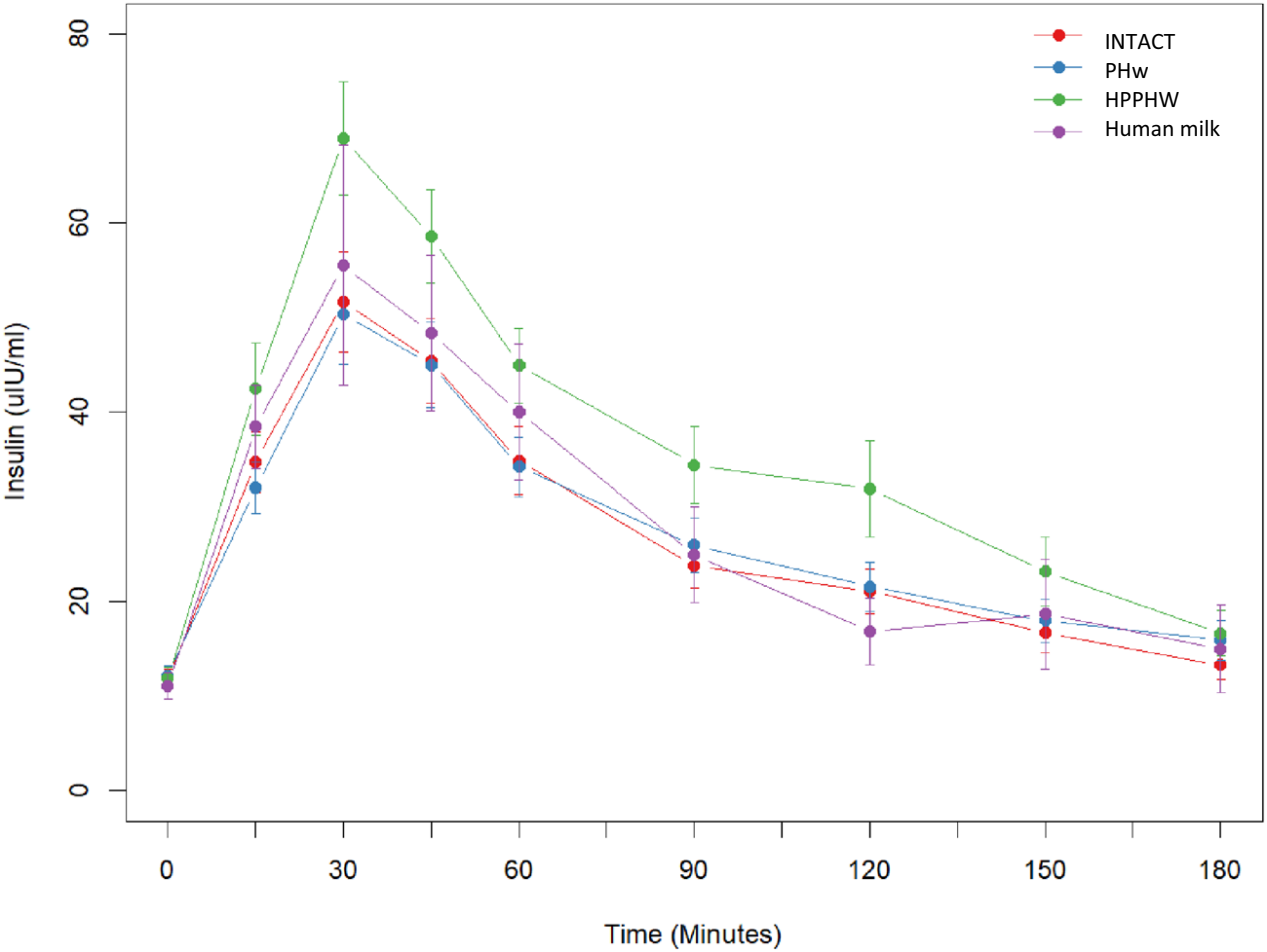
Insulin response to infant formulas and human milk overtime in healthy subjects from baseline and every 30 min for 180 min post-ingestion. Red: INTACT (*n* = 29), intact protein formula; Blue: PHw (*n* = 29), partially hydrolyzed whey protein formula; Green: HPPHW (*n* = 28), higher protein PHw formula; Violet: Human milk (*n* = 8). Data are mean ± SEM.

HPPHW had a significantly different time trend compared with INTACT, PHw, and HM (*p* < 0.001, <0.001, and 0.050, respectively from Likelihood Ratios (LR) test). HPPHW formula resulted in significantly higher insulin iAUC and Cmax response compared with both INTACT and PHw. The difference in iAUC between HPPHW vs. PHw was 49% (90% CI 30–68%, *p* < 0.001) and between HPPHW vs. INTACT was 51% (90% CI: 32–70%, *p* < 0.001). The iAUC for INTACT and PHw were close to HM. Cmax values were in line with the aforementioned findings for iAUC and can be found in Table 3. Changes in insulin over time in response to the infant formulas and HM can be seen in Fig. 2.

### Glucose

**Fig. 3:**
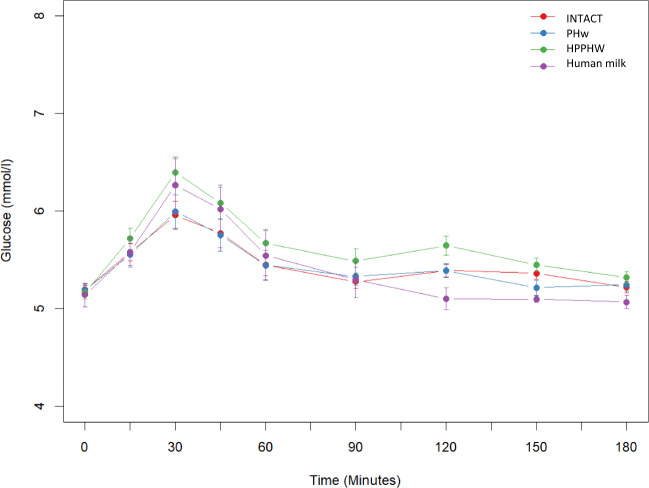
Glucose response to infant formulas and human milk overtime in healthy subjects from baseline and every 30 min for 180 mins post-ingestion. Red: INTACT (*n* = 29), intact protein formula; Blue: PHw (*n* = 29), partially hydrolyzed whey protein formula; Green: HPPHW (*n* = 28), higher protein PHw formula; Violet: Human milk (*n* = 8). Data are mean ± SEM.

Changes in glucose over time in response to the infant formulas and HM can be seen in Fig. 3. HPPHW showed a significantly different time trend compared with INTACT and PHw (*p* = 0.003 and *p* = 0.002, respectively, from LR tests). Results for iAUC and Cmax can be found in Table 3. Mean iAUC for HPPHW, HM, PHw and INTACT were 0.45, 0.40, 0.24, 0.24, respectively. Bioequivalence for iAUC and Cmax between PHw and INTACT was demonstrated. For iAUC, the difference between PHw and INTACT was −7.5% (90% CI −22.9 to 7.9%, *p* = 0.55). Mean Cmax for HPPHW, HM, PHw, INTACT was 6.25, 6.14, 5.86, 5.83 mmol/l, respectively. The difference between PHw and INTACT for Cmax was 0.16% (90% CI −1.7 to 2.05%, *p* = 0.84). HPPHW formula resulted in significantly higher blood glucose iAUC and Cmax response compared with both INTACT and PHw. iAUC: HPPHW vs. PHw showed a difference of 57% (90% CI: 32–83%, *p* < 0.001) and HPPHW vs. INTACT a difference of 50% (90% CI 23%, 76%, *p* = 0.003). The difference between HM vs. INTACT and HM vs. PHw were of a similar magnitude and no statistically significant differences were observed (Difference 42%, 90% CI 0.5–85%, *p* = 0.388 and 50% 90% CI 10–90%, *p* = 0.291, respectively). Results for Cmax mirrored these findings and can be found in Table 3.

### C-Peptide

Results for C-peptide can be found in Table 3. The time trend for C-peptide in response to HPPHW was significantly different compared with INTACT, PHw and HM (*p* < 0.001, <0.001, 0.041, respectively, from LR tests). HPPHW formula resulted in significantly higher C-peptide iAUC responses compared with both INTACT and PHw. HPPHW vs. PHw: Difference 54% (90% CI 33–76%, *p* < 0.001), HPPHW vs. INTACT: Difference: 55% (90% CI 33–78%, *p* < 0.001). Time trends for C-peptide were not statistically significantly different for INTACT and PHw compared to HM (*p* = 0.25 and 0.60, respectively, from LR tests).

### Glucagon

The time trend for glucagon in response to PHw was significantly different compared with INTACT and HPPHW (*p* < 0.001 and 0.032, respectively). Results for glucagon can be found in Table 3. The comparison between PHw and INTACT is best captured by Cmax where a difference of 15% was observed (90% CI 6–23%, *p* = 0.009). No significant differences in iAUC were observed in glucagon response between PHw and HPPHW.

### Triglycerides and free fatty acids

For triglycerides, HPPHW had a significantly different time trend compared with all other milk: INTACT, PHw, HM, (p = 0.009, 0.012, 0.012, respectively, from LR tests). Outcomes of triglyceride iAUC analysis can be found in Table 3. HPPHW formula resulted in significantly higher triglyceride iAUC compared with both PHw (difference 59% (90% CI 37–82%, *p* = 0.005)) and INTACT (difference: 56% (90% CI: 27–85%, *p* = 0.019)). Time trends for triglyceride values were not statistically significantly different for INTACT and PHw compared to HM (*p* = 0.71 and 0.94, respectively from LR tests).

No significant differences between time trends could be observed for free fatty acids (the smallest *p* value between the six comparisons was *p* = 0.19).

### Essential amino acids

Results for essential amino acids (EAA) iAUC can be found in Table 3. HPPHW resulted a significantly different time trend for EAA compared with PHw and HM (*p* = 0.014 and 0.009, respectively from LR tests). HPPHW formula resulted in significantly higher EAA iAUC compared with PHw (difference 74%, 90% CI 39–108%, *p* = 0.003) and HM (difference: 65%, 90% CI: 29–102%, *p* = 0.012). Time trends for EAA were not statistically significantly different for INTACT and PHw compared to HM (*p* = 0.50 and 0.92, respectively, from LR tests).

### Active GLP-1

No statistically significant differences between time trends could be observed for GLP-1.

## Discussion

To our knowledge, this is the first study to compare the glycaemic and insulinemic responses to infant formula containing different types (intact whey protein vs. partially hydrolyzed whey protein) and quantities (1.87 g/100 kcal - 2.8 g/100 kcal) of protein and to compare these formulas with HM.

Breastfeeding is the optimal source of nutrition for infants, but recent surveys indicate that a large proportion of infants receive some formula during the first year of life^2^. Therefore, understanding the metabolic responses to infant formula is crucial. Several trials have demonstrated the role of specific PHw formulas in reducing the risk of atopic disease in at-risk infants during the first year of life^26^. However, half of the infants with allergy or atopic dermatitis in the first year of life had no prior family history of allergy; the use of PHw formulas in the general infant population has also proven to be efficacious in reducing the risk of developing atopic dermatitis^27^. In recent years, the metabolic response to partially hydrolyzed infant formula has been questioned based on the assumption that PHw formulas would result in a higher postprandial aminoacidemia and thus, greater insulin secretion by the pancreatic β-cells^28^, due to the fact that PHw protein induces more rapid gastric emptying and a higher release of plasma amino acids compared with intact protein^29,30^. Moreover, a 2016 consensus statement concluded that there was a lack of data related to the metabolic effects of PHw formulas^6^. Therefore, in order to begin to address this research gap and to investigate the metabolic effects of formula containing different forms and quantities of protein, the current study tested the effect of three infant formulas differing in protein form and quantity ((i) INTACT: intact protein 70/30 whey/casein, (ii) PHw: partially hydrolyzed 100% whey protein, and (iii) HPPHW: high-protein partially hydrolyzed 100% whey) in healthy adults and compared them to a HM reference subgroup. The overall objective of the study was to show that the metabolic responses to INTACT and PHw formulas were similar.

Formally, the study objective was met since INTACT and PHw were found to be similar when applying the bioequivalence criteria devised by the EMA. The 90% CI for the iAUC response of insulin and glucose was within the predefined EMA boundaries of −20 and +25%. Our findings support the work of Agosti et al.^20^, who found no significant difference in insulin and glucose responses between hydrolyzed vs. intact protein formula in preterm newborn infants^20^. Furthermore, our results provide evidence in support of a recent systematic review and expert consensus which concluded that there is no potential harm associated with the use of PHw vs. cow’s milk (intact) protein formula^31^, at least with respect to insulin and glucose responses.

In addition to protein type, the effect of protein quantity in early life is of great interest. The early protein hypothesis posits that a protein supply that exceeds metabolic requirements during early life increases circulating and tissue concentrations of insulinogenic amino acids, resulting in an increase in growth mediators (insulin, IGF-1), which leads to increased weight gain and adiposity with implications for later obesity development^32^. In support of this hypothesis, studies show that a high-protein intake in infancy (particularly milk protein) increases body weight, adiposity and long-term risk for obesity^33,34^. In the current study, we tested the effect of a high-protein PHw formula (2.8 g protein/100 kcal) compared to the INTACT (1.87 g/100 kcal) and PHw (1.96 g/100 kcal) formulas and a HM subgroup. We found a greater effect of the HPPHw on insulin, glucose, C-peptide, triglycerides and essential amino acids, potentially indicating a less favourable effect of the higher PHw protein load. We observed this effect in adults, though it is possible that a higher protein load might also exert less favourable effects on the aforementioned parameters in infants. Indeed, previous studies have shown that reducing the protein quantity of infant formula decreased the plasma concentrations of insulin and essential amino acids in infants^35,36^.

In the current study, we provided protein in the range of 7–12 g. Using greater protein loads (21.6 g), Claessens et al^19^. found no difference in insulin response between intact whey protein vs. whey protein hydrolysate in non-obese men, thus confirming our findings. In the same study, significant differences in insulin response were observed only in response to a higher intact whey protein load (28.8 and 43.2 g), but not whey protein hydrolysate. In contrast, Power et al.^37^ demonstrated greater insulin concentrations following ingestion of 45 g of a whey protein hydrolysate vs. the same amount of intact whey protein. The findings from the three studies suggest that, in adults, for quantities of protein up to ≈22 g/d there appears to be no significant difference in insulin responses between intact and hydrolyzed whey proteins; however, differences may become apparent at protein quantities of greater than ≈28 g. However, it remains unclear whether the greater insulin response to the higher protein load is primarily driven by intact or hydrolyzed whey protein and this requires further substantiation. Differences between our study and the two aforementioned studies^19,37^ may also be related to the fact that our milk contained lipids and carbohydrates in addition to protein, which may have influenced the insulin response. Indeed, it has been shown that a combination of macronutrients (lipids, carbohydrate and protein) added to a carbohydrate-containing food augmented the insulin AUC response compared to when protein alone was added^38^, suggesting that the combination of macronutrients in our milk may have influenced glycaemic responses.

**Fig. 4:**
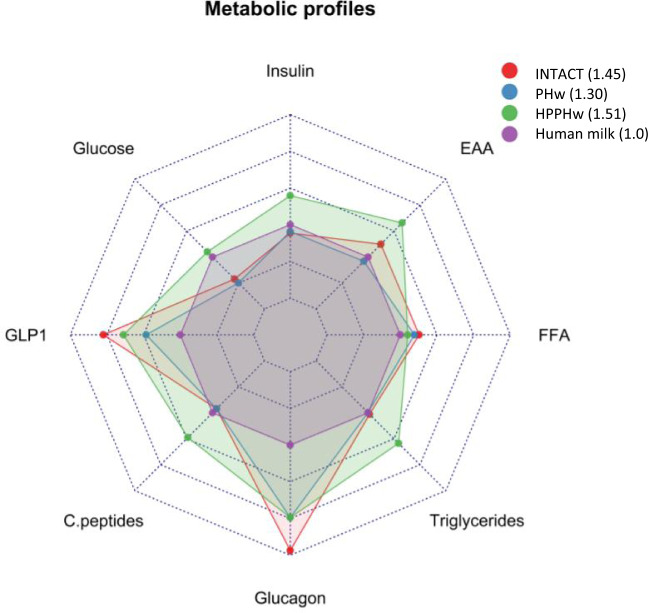
Metabolic profiles of the infant formulas with respect to human milk (HM) based on iAUC (and negative AUC for free-fatty acids only). Red: INTACT (*n* = 29), intact protein formula; Blue: PHw (*n* = 29), partially hydrolyzed whey protein formula; Green: HPPHW (*n* = 28), higher protein PHw formula; Violet: Human milk (*n* = 8). The differences compared with HM were estimated by permutation tests. The scales of the radar plot range from 0 to 250%. The numbers in parentheses in the legend (1.45, 1.30, 1.51) indicate the average differences compared to HM, which is considered the reference as assigned a value of 1.0.

The direction of the results compared to the HM reference subgroup is illustrated in the radar plot in Fig. 4. Our finding of no significant difference in insulin or glucose responses between HM and PHw and INTACT formulas is supported by the work of Wright et al.^12^ who investigated the effect of HM compared with typical commercial infant formula and found no difference in postprandial glycaemia or insulinaemia. They suggest that a potential explanation for the lack of significant difference may be that both infant formula and breast milk are low GI (i.e. ≤55) and they suggest that although there are compositional differences between breast milk and infant formula, the energy and macronutrient contents of both were similar, which may account for this lack of difference in glycaemic responses.

Glucagon plays an important role in increasing blood glucose concentrations and thus, counteracts the actions of insulin. In the current study, glucagon was significantly higher in response to INTACT vs. PHw. In contrast to our findings, Claessens et al.^19^ found no difference in glucagon response between 21.6 g of intact and hydrolyzed whey protein, except when protein load was increased, in which case hydrolyzed whey protein increased glucagon AUC significantly more than intact whey protein. In another study, whey peptide hydrolysates (≈15 g/d) were compared against pea protein hydrolysates and complete cow’s milk protein and no difference was observed in terms of glucagon response^18^. Differences between studies may relate to the greater quantity of protein provided in these studies, as well as the fact that unlike the other studies, our protein was provided in the matrix of infant formula.

This is the first study to compare the metabolic effects of partially hydrolyzed protein vs. intact protein and to have a HM reference group. However, one important limitation is that the study could not be performed in infants due to the ethical constraints related to repeated blood sampling in infants. Nonetheless, although the metabolic responses to each intervention in terms of absolute values may be different between adults and infants, the relative difference between tested products is not likely to be very different and differences between the treatments are likely to be in the same direction. The volume of 600 ml used in this study is greater than the typical meal intake of young children (≈200 ml for age 3–5 months), however, this volume was chosen to provide 350 kcal, which represents a small meal in adults. There were a limited number of subjects in the HM group as comparisons with HM were not the main objective of the study and HM was not available for all subjects. As a result, the HM group included only 8 subjects, whereas the formula groups had 29 subjects. Thus, comparisons with the HM group are lacking power and need to be interpreted with caution. In addition, future studies should include a measure of the rate of gastric emptying, which may provide further insight into the metabolic responses to infant formula containing different types and quantities of protein.

## Conclusion

The current study found no significant differences in glucose or insulin responses between a formula containing partially hydrolyzed whey protein and a formula containing intact whey protein or HM, although the comparison with HM was not adequately powered to detect a difference. The HPPHw formula (2.8 g/100 kcal) induced greater glucose and insulin responses compared to the other interventions and although these results pertain to adults and thus require verification in infants, it is possible that similar findings might be observed in infants, which could conceivably align with the early protein hypothesis. Moreover, these apparent anabolic effects of the high-protein formula might be desirable for catch-up growth in preterm or low birth weight infants. Understanding the metabolic responses to infant formula containing different types and quantities of protein is important, not only to help inform the development of infant formula that more closely resembles HM, but to help understand potential imprinting effects and the associated long-term impact of infant formula on metabolic health.
